# Evidence-based approaches for Empty Nose Syndrome management: a systematic review highlighting current treatments and future directions

**DOI:** 10.3389/fbioe.2026.1778266

**Published:** 2026-04-08

**Authors:** Giulia Galaverni, Davide Adamo, Anjana Chathanchirappattu Raj, Vincenzo Giuseppe Genna, Graziella Pellegrini

**Affiliations:** 1 Department of Life Sciences, Centre for Regenerative Medicine “Stefano Ferrari”, University of Modena and Reggio Emilia, Modena, Italy; 2 Department of Surgery, Medicine, Dentistry and Morphological Sciences with Transplant Surgery, Oncology and Regenerative Medicine Relevance, Centre for Regenerative Medicine “Stefano Ferrari”, University of Modena and Reggio Emilia, Modena, Italy; 3 Holostem S.r.l., Modena, Italy

**Keywords:** empty nose syndrome, ENS reconstructive approach, ENS treatment, inferior meatus augmentation, systematic review

## Abstract

**Background:**

Empty nose syndrome (ENS) is an iatrogenic condition that develops following turbinate surgery. ENS presents with paradoxical nasal obstruction, sensation of suffocation, nasal dryness, accompanied by a substantial psychological burden, resulting in a high suicide rate and a severely diminished quality of life. Available treatments for ENS are often ineffective, underscoring the need for innovative interventions. This systematic review provides a comprehensive overview of the current therapeutic strategies for ENS and evaluates their reported efficacy.

**Methods:**

Following PRISMA guidelines, a systematic search was conducted across PubMed, Embase, Scopus, Web of Science, ClinicalTrials.gov, and EudraCT in August 2025. Of 788 identified studies, 44 met the inclusion criteria: English language, full-text availability, human subjects, primary focus on ENS treatment, and classification as research articles or case reports. Study characteristics were organized into macro-themes - study type, population, therapeutic approach, follow-up, outcomes, adverse events, and vascularization strategies - and related micro-themes.

**Results:**

The majority of studies were case series (30), while fewer studies consisted of case reports (8), case–control (3), cohort (3), and randomized controlled trials (1). Sample sizes were limited, with a predominance of male participants. Implant-based interventions were the most common (70.5%), followed by injection-based and neurostimulation approaches, while pharmacological and/or cognitive therapies were explored in three multimodal designs. Most studies had short-to mid-term follow-up, with 61.4% lasting less than 1 year. Outcomes were mainly assessed via questionnaires (75%) and clinical evaluations (50%), whereas biological analyses were rarely performed (9.1%). Postoperative adverse events were analysed in 64% of studies and predominantly occurred within the first month.

**Conclusion:**

This review highlights that ENS treatment research is largely dominated by descriptive and observational studies, reflecting the current limited evidence. The heavy reliance on subjective, questionnaire-based outcomes further increases the risk of bias, emphasizing the need for a deeper understanding of ENS pathogenesis and the integration of objective biological measures as outcome metrics. Building on the most promising approaches, future studies should focus on conducting larger randomised controlled trials to develop standardised, evidence-based treatment protocols for patients with ENS, involving different strategies to control the pathology.

## 1 Introduction

Empty Nose Syndrome (ENS), first described in 1994, is an iatrogenic condition that develops after nasal surgery, typically partial or total inferior turbinectomies (Hong and Jang, 2016; Gordiienko et al., 2021). ENS is characterized by paradoxical nasal obstruction despite an objectively patent nasal airway. Symptoms vary and include: nasal dryness, crusting, bleeding, a sensation of suffocation, and inadequate lung inflation (Houser, 2006; Chhabra and Houser, 2009; Scheithauer, 2010; Coste et al., 2012). ENS is also associated with poor sleep quality (Huang A. N. et al., 2022; Huang et al., 2023), difficulty concentrating, anxiety, depression, panic attacks, and, in many cases, suicidal thoughts (Huang et al., 2022b). These manifestations severely impact quality of life and cause substantial psychological distress. The pathogenesis of ENS remains unclear (Kanjanawasee et al., 2022; Esteban-Ortega et al., 2025), consequently some otolaryngologists still question its existence (Png et al., 2023). However, numerous studies based on virtual surgery and computational fluid dynamics (CFD) demonstrated a radical reduction in air-flow resistance and air conditioning coupled with sensorineural dysfunction (Di et al., 2013; Dayal et al., 2016; Balakin et al., 2017; Thamboo et al., 2017; Malik et al., 2019; Maza et al., 2019). Histological analyses have also revealed deep mucosal changes, including epithelium remodeling and thermoreceptor downregulation, which contribute to ENS symptomatology (Wu et al., 2021). ENS diagnosis relies mainly on symptom-based questionnaires, such as Sino-Nasal Outcome Test (SNOT), Empty Nose Syndrome 6-Item Questionnaire (ENS6Q), and Nasal Obstruction and Septoplasty Effectiveness scale (NOSE) (Thamboo et al., 2017; Amanian et al., 2021), nasal endoscopy, imaging like Computed Tomography (CT) scan or Magnetic Resonance Imaging (MRI), Cotton-test and nasal airflow/resistance tests (Thamboo et al., 2017). Despite these tools and ongoing efforts to identify objective specific clinical, radiographic, and endoscopic findings (Dholakia et al., 2024), ENS remains underrecognized (Aguirre-Peña et al., 2025). While the true prevalence is uncertain, some reports suggest an occurrence rate of 16%–23% after turbinate surgery, with symptoms developing even months or years postoperatively (Coste et al., 2012; Park et al., 2024). Prevention is the first-line strategy to reduce the risk of ENS. Conservative surgical techniques may help reduce incidence (Gotlib et al., 2020; Çelik et al., 2024), though even modern methods have been associated with its onset (Kim et al., 2021; Torabi et al., 2024). Besides, the rising number of nasal surgeries for aesthetic purposes or to manage chronic rhinitis is contributing to the increase in ENS cases (Park et al., 2024). Current treatments primarily focus on symptom management through nasal moisturizers, saline rinses, and medications, which often provide limited or no significant relief (Gill et al., 2019). Emerging strategies include reconstructive surgery, regenerative medicine approaches, and psychological or cognitive therapies (Gordiienko et al., 2021; Hussain et al., 2024; Kim and Hwang, 2024; Aguirre-Peña et al., 2025). However, none have yet achieved the standardization, reproducibility, and long-term safety and efficacy required for routine clinical implementation (Aguirre-Peña et al., 2025). The lack of unified protocols and objective metrics further complicates comparison across treatment strategies. This review systematically summarizes current experimental treatments for ENS, classifying them by approach type and other macro-themes. It also examines factors affecting study reliability, such as sample size and follow-up duration, evaluates treatment effectiveness, highlights promising strategies, and discusses potential complications. Particular attention is given to reconstructive surgery elements, such as vascularization and innervation, which are often neglected in the existing literature.

## 2 Methods

This study was performed in accordance with the Preferred Reporting Items for Systematic Reviews and Meta-analysis (PRISMA) checklist and statement recommendations (Page et al., 2021).

### 2.1 Search strategy and selection process

A comprehensive search was conducted across PubMed, Embase, Scopus, Web of Science, ClinicalTrials.gov and EudraCT to retrieve all relevant data on ENS treatment. The search, performed on 28 August 2025, imposed no time restrictions. The search was carried out using the following keywords: “Empty Nose Syndrome”, “Turbinate reconstruction”, “Empty Nose Syndrome treatment”, and “Secondary atrophic rhinitis”. Two authors (GG and DA) independently reviewed the retrieved studies, assessing titles and abstracts to identify potentially relevant articles. When abstracts lacked sufficient information, the full texts were examined. Duplicate records were excluded. Full texts of the selected studies were then evaluated for eligibility against predefined inclusion and exclusion criteria. In cases of disagreement, consensus was reached through discussion with a third author (ACR). The inclusion criteria were as follows: human studies; classification as research articles (including case series, cohort studies, case–control studies and randomized controlled trials) or case reports; articles published in English; and a primary focus on ENS treatment. Any treatment strategy for ENS - including surgical and psychosomatic interventions - was considered. Studies addressing conditions other than ENS or not focused on ENS treatment, as well as review articles, letters, editorials, commentaries, books and preprints, were excluded. When overlapping data were found, the most comprehensive article was included for analysis.

### 2.2 Data collection process and data items

The studies that met our inclusion criteria were independently assessed by three authors (GG, DA, and ACR). A structured Excel (Microsoft 365, Version 16.102) data collection sheet was developed to extract data from the full texts. The study characteristics were analyzed and subdivided into macro-themes and micro-themes (study domains). Macro-themes covered study population, therapeutic approaches, follow-up duration, outcome measures, outcome measurement, and adverse events. The micro-themes explored specific aspects of implant-based approaches (type of material, implant dimensions, and site of implantation) and injection-based approaches (injected material, injection site, injected volume, N. of injections).

For each included study, all available results relevant to the respective domains were extracted (e.g., for all measures/measurements, follow-up time points, analyses). In studies reporting patients with different pathologies, only data pertaining to ENS patients were included. Any discordance was resolved through discussion among the authors. Missing or unclear information was documented and considered in the risk of bias assessment.

### 2.3 Study risk of bias assessment

Considering the heterogeneity of study designs among the eligible and included studies, they were categorized as case reports, case series, cohort studies, case–control studies and randomized controlled trials, and the risk of bias was assessed using design-specific tools. The Joanna Briggs Institute (JBI) critical appraisal checklist was applied to case reports, while the National Institutes of Health (NIH) guidelines were used for all other study designs. For each study, the risk of bias was assessed by two independent authors (AD and ACR). In case of discrepancies, consensus was reached with the involvement of a third author (GG).

## 3 Results

### 3.1 Study selection

**FIGURE 1:**
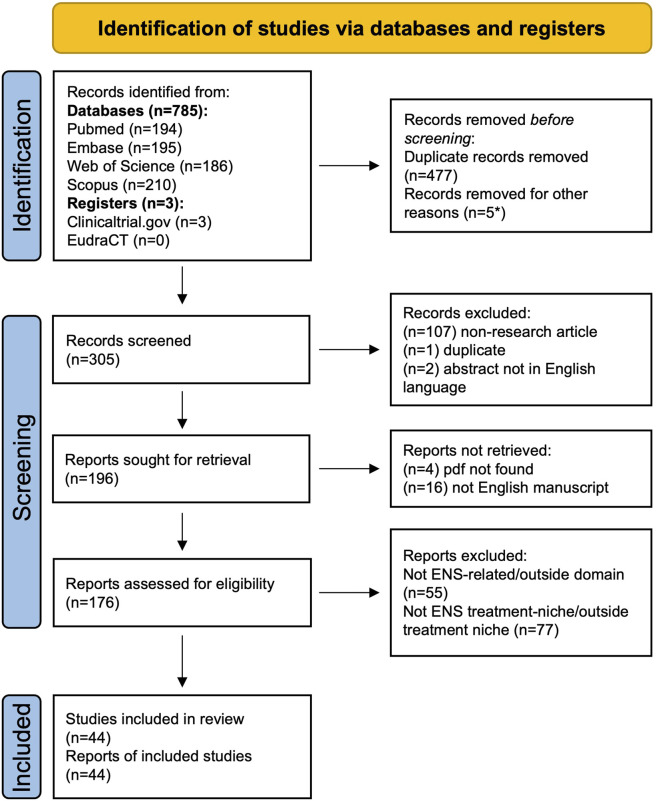
PRISMA flowchart of study selection process. PRISMA flowchart illustrating the process of identification, screening, and selection of the studies included in the review. *Records excluded from databases (n = 2) for abstract and manuscript not retrievable; records excluded from registries (n = 3) for results not available or insufficient information. This work is licensed under CC BY 4.0 (Page et al., 2021).

The PRISMA guidelines were followed to report the selection of the studies included in this systematic review. Research using predefined keywords across four databases (PubMed, Embase, Web of Science, Scopus) and two registers (ClinicalTrials.gov, EudraCT) identified 788 studies. Title and abstract screening led to 305 records, of which 176 full texts were assessed for eligibility. Ultimately, the search strategy yielded 44 articles that met the inclusion criteria (Figure 1; Table 1).

**TABLE 1:** Summary of the included studies.

Study	Study design	Type of approach	N°. of patients	Treatment	Follow-up (months)	Outcome measures	Results	Complications/adverse events
Bastier et al. (2013)	Prospective case-series	Implantation	5 (3M; 2F)	A submucoperiosteal pocket along each lateral nasal wall was created by bilateral oral mucosa incision. Two layers of pure nonporous ß-tricalcium phosphate (ß-TCP) were inserted per side and secured with suture	13.5 (8.2–21)	Questionnaire: NOSE, RhinoQoL Clinical test: CT scan, endoscopy	NOSE: 90 vs*.* 5 (p < 0.05) RhinoQoL: Frequency 18.7 vs*.* 81.2 (p = 0.05); Bothersomeness 30 vs*.* 81 (p = 0.05); Impact 62.5 vs*.* 8.3 (p = 0.01) CT-scan and endoscopy evaluation showed reduced nasal cavity volume	20% of patients experienced implant extrusion (6 days post-op)
Bastier et al. (2016)	Prospective case-series	Implantation	14 (5M; 9F)	Two pure nonporous ß-tricalcium phosphate (ß-TCP) implants were inserted along the lateral wall (see Bastier et al., 2013)	19.4 (3.6–49)	Questionnaire: NOSE, RhinoQoL Clinical test: Endoscopy	NOSE: 73.93 vs*.* 34.64 (p < 0.05) RhinoQoL: Frequency 44.64 vs*.* 34.82 (p = 0.007); Bothersomeness 43.6 vs*.* 70.7 (p = 0.005); Impact 59.9 vs*.* 27.2 (p = 0.001) Endoscopy evaluation showed reduced nasal cavity volume	7% of patients experienced implant exposure (7months post-op.), 7% implant misplacement and 7% decreased comfort (24months post-op)
Bi and Zhou (2016)	Retrospective case report	Acupuncture	1 (M)	Acupuncture needles were inserted in multiple pressure points with reinforced and/or reduced manipulation	1	Other: Patients’ subjective report	Symptoms relieved	Headache (immediately post-int. and lasted for 6 days)
Borchard et al. (2019)	Prospective case-series	Injection	14 (10M; 4F)	Carboxymethylcellulose gel (Prolaryn®) gel was injected in multiple sites of the patient’s anterior-inferior lateral nasal wall to increase nasal soft tissue bulk. Injection sites varied based on individual cotton testing results and patient anatomy	3	Questionnaire: SNOT-22, ENS6Q, PHQ-9, GAD-7	SNOT-22: 50.3 vs*.* 29.3 (1w, p = 0.01), 35.5 (1 month, p = 0.04), 39.3 (3 months, p > 0.05) ENS6Q: 20.8 vs*.* 10.5 (1w, p < 0.0001), 13.7 (1 month, p = 0.002), 15.5 (3 months, p > 0.05) PHQ-9: 11.6 vs*.* 6.6 (1w, p = 0.01), 7 (1 month, p = 0.004), 7.9 (3 months, p > 0.05) GAD-7: 8.6 vs*.* 5.4 (1w, p > 0.05), 4.9 (1 month, p = 0.02), 5 (3 months, p = 0.02)	No major complications reported; transient sensation of pressure at site of injection in some patients
Buiret (2024)	Retrospective case-series	Injection	11 (6M; 5F)	Adipocytes extracted from autologous adipose tissue, collected via umbilical puncture, were injected into the head and body of the inferior turbinate, lateral nasal wall and nasal floor	1	Questionnaire: ENS6Q	ENS6Q: 16 vs*.* 9 (p = 0.0058)	No complications reported
Cameron et al. (2025)	Retrospective case-series	Implantation	16 (N/A)	Small strips of purified bovine derived collagen matrix (BDCM) were packed into a submucosal pocket. The location and quantity of the graft were guided by cotton test results	mean 9.3	Questionnaire: ENS6Q Clinical test: Endoscopy	ENS6Q: 17.5 vs*.* 9.7 (4w, p = 0.0001), 11.2 (9 months, p = 0.602) Endoscopic evaluation confirmed low resorption rate of implant	No major complications reported
Chang (2024)	Prospective case-series	Implantation	2 (M)	Platelet-rich fibrin scaffolds embedded with diced cartilage grafts were used to fill the submucosal pocket created below the inferior turbinate remnant. The incision wound was covered with Hemopatch for coagulation effect and haemostasis	12	Questionnaire: ENS6Q Clinical test: Endoscopy	ENS6Q: 20.5 vs*.* 6 (12 months) Endoscopic evaluation confirmed stable implantation of the graft	No complications reported
Chang et al. (2021)	Prospective cohort	Implantation	40 (31M; 9F)	Multiple pieces of Medpor® sheet were used to form an ideal contour of submucosal implantation. The implant volume was based on patient anatomy	6	Questionnaire: SNOT-25, ENS6Q, BAI, BDI-II, SS-12 Other: Olfaction rating	SNOT-25: (p < 0.05) ENS6Q: hyposmia (p < 0.05) normosmia (N/R) BAI and BDI-II: hyposmia (p < 0.05) normosmia (N/R) SS-12: hyposmia (3 months, p = 0.023; 6months, p = 0.031) normosmia (N/R) Olfaction improvement post implantation; age having significant correlation with improvement	N/R
Dholakia et al. (2021)	Prospective case-series	Implantation	17 (8M; 9F)	Packaged, decellularized, and irradiated segments of cadaveric rib cartilage were carved into two pill-shaped implants of defined size and then carefully placed into the submucosal pockets of the bilateral inferior meatuses	12	Questionnaire: SNOT-22, ENS6Q, GAD-7, PHQ-9	SNOT-22: 46.8 vs*.* 30.6 (1 month, p = 0.0021), 32.4 (3 months, p = 0.0227), 30.9 (6 months, p = 0.0004), 29.4 (12 months, p = 0.0025) ENS6Q: 18.7 vs*.* 7.4 (1 month, p < 0.0001), 8 (3 months, p < 0.0001), 7.8 (6 months, p < 0.0001), 8.4 (12 months, p = 0.0003) GAD-7: 7 vs*.* 4.8 (1 month, p = 0.5155), 5.4 (3 months, p > 0.999), 6 (6 months, p > 0.999) PHQ-9: 9.4 vs*.* 6.1 (1 month, p = 0.5725), 6.6 (3 months, p > 0.9313), 7.3 (6 months, p > 0.999)	70% of patients experienced mild crusting and edema (1 month post-op.); 5.8% of patients experienced neck stiffness, emesis, eye swelling, headache (immediately after IMAP) treated with medication; 5.8% of patients experienced mild epistaxis (N/R) resolved with in-office cauterization
Fu et al. (2021)	Prospective case-series	Implantation	43 (37M; 6F)	Multiple pieces of Medpor® grafts were used to form an ideal contour of submucosal implantation. The implant volume was based on patient anatomy	12	Questionnaire: SNOT-25, ENS6Q, BAI, BDI-II Clinical test: Endoscopy, serum hs-CRP level	SNOT-25: p < 0.001 ENS6Q: p < 0.001 BAI: p < 0.001 BDI-II: p < 0.001 post-op hs-CRP levels decreased in depressed (p = 0.025) and anxious (p = 0.005) patients	N/R
Gwak et al. (2024)	Retrospective case-series	Nasal plug	20 (14M; 6F)	A 3D-printed nasal plug of polyurethane elastomer and hardener blend, with head and external loop made of shape memory polymer was placed into the nasal cavity; the head portion fills the defect, touching the turbinate and lateral wall mucosa	10 (2.1–27.5)	Questionnaire: ENS6Q Other: Patients’ compliance and satisfaction	ENS6Q: 19.6 vs*.* 6.8 (30 min after nasal plug positioning) Patients’ compliance and satisfaction: 4% excellent, 8% good, 5% moderate, 3% poor	Out of 20 patients, 65% experienced nasal plug displacement, 60% cosmetic concerns, 20% foreign body sensation, 15% runny nose; 5% pain, 5% n = 1 itching Approach discontinued in a few cases
Gwak and Jang, 2026	Retrospective case-series	Implantation	32 total, 20 (16M; 4F) clinically analysed	Autologous costal cartilage was trimmed into a boat-shaped graft to fit the defect and inserted into a submucosal pocket, with 3–4 mm space between nasal wall and septum. If required, additional costal cartilage or autologous fat was used for fine-tuning implant’s volume	61.2 ± 28.8	Questionnaire: ENS6Q	ENS6Q: 17.5 vs*.* 9.1 (p < 0.001)	Out of 32 total patients, 56.3% experienced complications: 34.4% nasal obstruction; 9.4% discharge and postnasal drip; 6.3% headache; 6.3% costal wound complication; 3.1% frequent epistaxis; 3.1% chronic nasal pain; 3.1% foreign body sensation; 3.1% synechiae; 9.4% flap laceration during the surgery. Adverse events managed by graft removal (n = 3) or reduction (n = 1)
Hassan et al. (2022)	Prospective case report	Implantation	1 (M)	A non-humidified ceramic glass implant (GlassBONE™) was sculpted and placed in a submucoperiosteal space	4	Clinical test: Endoscopy	Endoscopic aspect was satisfying with long-term improvement of nasal obstruction	No complications reported
Hosokawa et al. (2025)	Prospective case-series	Implantation	9 (8M; 1F)	Autologous dermal fat was implanted to fill a mucosal pocket	3	Questionnaire: ENS6Q Clinical test: CT-scan, endoscopy	ENS6Q: 20 vs*.* 9.44 (1w, p = 0.0076), 5.56 (1 month, p = 0.0075), 5 (3 months, p = 0.0076) Clinical tests show reduction in nasal cavity volume	No complications reported
Houser (2006)	Prospective case report	Implantation	1	Acellular dermis (AlloDerm) was implanted in a septal submucoperiosteal space, further augmented with Cymetra implant 3months later	3	Clinical test: CT-scan, endoscopy Other: Patients’ subjective report	Patient reported 40% improvement in symptoms	N/R
Houser (2007)	Prospective case-series	Implantation	8 (7M; 1F)	Acellular dermis (AlloDerm) was used to fill a submucosal pocket. Implantation volume varied based on individual cotton test results and patient anatomy	26,6 (6–48)	Questionnaire: SNOT-20 Clinical test: Histopathology	SNOT-20: 58.3 vs*.* 32.4 (6 months, p < 0.001) Histological analysis of the implant showed signs of integration with small blood vessels and fibroblasts-embedded collagen	No complications reported
Hsueh et al. (2023)	Prospective case-series	Implantation	35 (26M; 9F)	Small pieces of Medpor® were implanted in a submucosal pocket.	12	Questionnaire: SNOT-25, ENS6Q, BAI, BDI-II	SNOT-25: p < 0.0001 ENS6Q: (p < 0.0001) BAI: (p < 0.0001) BDI-II: (p < 0.0001)	N/R
Hsueh et al. (2025)	Prospective case-series	Implantation	39 (33M; 6F)	A submucosal pocket was filled with small pieces of Medpor® (same as Hsueh et al., 2023)	6	Questionnaire: ENS6Q, BAI, BDI-II, PSQI, EpSS Clinical test: Polysomnography	ENS6Q: (p < 0.001) BAI: (p < 0.001) BDI-II: (p < 0.001) PSQI: (p < 0.001) EpSS: (p < 0.01)	No major complications reported
Huang A.N. et al. (2022)	Prospective case report	Implantation	1 (N/R)	Expanded polytetrafluoroethylene (Gore-Tex®) was implanted at the submucosal floor	(N/R)	Clinical test: CT-scan, CFD Other: Patients’ subjective report	CT-scan CFD confirmed nasal cavity volume reduction, redistribution of airflow, higher airflow fraction, enhanced heat and water vapor fluxes, enhanced nasal air conditioning ability. Subjective questionnaire confirmed symptoms improvements	N/R
Huang et al. (2019)	Prospective case-series	Implantation	68 (51M; 17F), analyzed 39 (25M; 14F)	Medpor® were implanted in a submucosal pocket.	6	Questionnaire: SNOT-25, BAI, BDI-II Clinical test: Endoscopy, serum IgE level	SNOT-25: 62.9 vs*.* 63.3 BAI: 17.7 vs. 21.2 (p = 0.007) BDI-II: 19.3 vs. 21.3 serum IgE level did not change significantly	N/R
Huang et al. (2021)	Prospective case-series	Implantation	54 (36M; 18F), analyzed 6 months 45 (29M; 16F); analyzed 12 months 38 (25M; 13F)	Medpor® were implanted in a submucosal pocket.	(3–12)	Questionnaire: SNOT-25, BAI, BDI-II	SNOT-25: 54.9 vs*.* 32.9 (3 months), 30.2 (6 months), 29.1 (12 months) p < 0.001 BAI: 20.7 vs*.* 8.2 (3 months), 8 (6 months), 7.8 (12 months), p < 0.001 BDI-II: 20.7 vs*.* 8.2 (3 months), 8 (6 months), 7.8 (12 months), p < 0.001	N/R
Huang et al. (2023)	Prospective case-series	Implantation	74 (55M; 19F)	Medpor® were implanted in a submucosal pocket.	(6–36)	Questionnaire: SNOT-25, ENS6Q, BAI, BDI-II	SNOT-25: 65.1 vs*.* 32.4 (6 months, p < 0.001) ENS6Q: 15.6 vs*.* 7.6 (6 months, p < 0.001) BAI: 19.4 vs*.* 10 (6 months, p < 0.001) BDI-II: 20.3 vs*.* 8.5 (6 months, p < 0.001)	6.8% of patients experienced implant exposure and infection. Adverse events managed by reintervention
W Iqbal and Gendeh (2007)	Prospective case report	Pharmacological	1 (M)	Steroid nasal sprays, mucolytics, oral antibiotics, Sterimar and Singulair (Montelukast) were used for symptomatic relief	Unclear	Other: Patients’ subjective report	Intermittent symptom relief with long-term medical therapy of nasal steroids, antihistamines, leukotriene receptor antagonist	N/R
Jang et al. (2011)	Retrospective case-control	Implantation	12 (8M; 4F)	Septal, conchal, autologous, or homologous costal cartilage was used to fill a submucosal pocket.	12.8 (6–27)	Questionnaire: Not validated patients’ subjective questionnaire using VAS	Excessive airflow: 8.86 vs*.* 2.71 (p = 0.027) Nasal obstruction: 8 vs*.* 3 (p = 0.028) Nasal or facial pain: 8.33 vs*.* 2.33 (p = 0.042) Rhinorrhoea or post-nasal drip: 7 vs*.* 4.67 (p = 0.180) Headache: 8.5 vs*.* 0 (p = 0.180) 25% of patients rated as under corrected	N/R
Jiang et al. (2013)	Prospective case-series	Implantation	19 (15M; 4F)	One to four pieces of Medpor® were implanted in a submucosal pocket to replace missing tissue. Implantation sites varied based on individual needs	12	Questionnaire: SNOT-20 Clinical test: CT-scan, endoscopy, acoustic rhinometry, MCC	SNOT-20: 50.1 vs*.* 22.6 (3 months, p = 0.037), 20.4 (6 months, p = 0.007), 37.7 (12 months, p = 0.736) Endoscopic evaluation and CT images confirmed stable implantation Acoustic rhinometry assessment showed significant improvement in nasal volume, nasal resistance and minimum cross-sectional area. No statistically significant MCC improvement	5.2% of patients experienced partial Implant expulsion (6 months post-op)
Jiang et al. (2014)	Prospective case-series	Implantation	24 (18M; 6F)	To replace the missing turbinate tissue 1 to 4 pieces of Medpor® were implanted in a submucosal pocket. Implantation sites varied based on individual needs (same as Jiang et al., 2013)	12	Questionnaire: SNOT-25	SNOT-25: 68.31 vs*.* 49.6 (3 months, p = 0.045), 30.69 (6 months, p < 0.001), 27.75 (12 months, p < 0.001)	No complications reported
Jung et al. (2013)	Retrospective case-control	Implantation	31 (22M; 9F)	Two treatments groups: i. Conchal cartilage group (n = 17): autologous cartilage was rolled into a spherical kidney-shaped structure and implanted into a submucosal pocket. ii. Costal cartilage group (n = 14): autologous costal cartilage (n = 8) and homologous costal cartilage (n = 6) were carved into round-shape structures and implanted into a submucosal pocket	(6–12)	Questionnaire: SNOT-25 Clinical test: Endoscopy, CT-scan	SNOT-25: costal cartilage group (p < 0.05); conchal cartilage group (p < 0.05) Endoscopy and CT-scan examinations indicated good mucosal healing and stable implantation	No complications reported
Kim et al. (2018)	Prospective case-series	Injection	17 total, 10 analysed (7M; 3F)	High-density stromal vascular fraction was extracted from autologous abdominal fat and injected into the remnant inferior turbinate	6	Questionnaire: SNOT-25 Clinical test: Nasal secretion analysis	SNOT-25: 70.1 vs*.* 62.4 (p > 0.05) Nasal secretion analysis showed IL-1 β and IL-8 levels were significantly decreased after injection (p < 0.005)	10% of patients experienced seroma treated via ultrasound-guided aspiration
Le Bon et al. (2020)	Prospective case-series	Neurostimulation	14 total (7M; 7 F), 7 analysed	To stimulate trigeminal receptor TRPM8, patients were subjected to an intranasal trigeminal training consisting of three-times daily levomenthol and eucalyptol inhalations for at least 30 days	1.4 (1–2)	Questionnaire: SNOT-22, NOSE, ENS-6Q	SNOT-22: 63.7 vs*.* 50.7 (1.4 months, p = 0.028) NOSE: 12.4 vs*.* 9.8 (1.4 months, p = 0.027) ENS-6Q: 17.1 vs*.* 13.8 (1.4 months)	N/R
Lee et al. (2023)	Prospective case-series	Injection	2 (M)	Ten to twelve doses of autologous platelet-rich plasma were injected into the remnant inferior turbinate	3.5 (2–5)	Questionnaire: SNOT-22, NOSE Clinical test: Endoscopy	SNOT-22: Case 1: 50 vs*.* 49 (4 months), 37 (5 months) Case2: 21 vs. 6 (1 month), 4 (2 months) NOSE: Case 1: 11 vs*.* 7 (4 months), 8 (5 months) Case2: 3 vs. 0 (1 month), 8 (2 months) Endoscopic examination showed decreased nasal cavity volume and resolved mucosal dryness	N/R
Lee et al. (2016)	Prospective cohort	Implantation	20 (12M; 8F)	Multiple pieces of Medpor® (n = 13) or septal bone graft, when available (n = 7), were submucosally implanted on the nasal floor	12	Questionnaire: BAI, BDI-II Clinical test: Endoscopy	BAI: 19 vs*.* 6.8 (p < 0.001) BDI-II: 24.4 vs*.* 6.3 (p < 0.001) Endoscopic examination showed decreased nasal cavity volume and improved mucosal healing	N/R
Lee et al. (2018)	Retrospective case-control	Implantation	30 (19M; 11F)	Small pieces of Medpor® were implanted in a submucosal pocket within the inferior nasal wall (n = 14) or lateral nasal wall (n = 16)	12	Questionnaire: SNOT-22, BDI-II, BAI	SNOT-22: inferior nasal wall group (p = 0.002); lateral nasal wall group (p < 0.001) BDI-II: inferior nasal wall group (p = 0.031); lateral nasal wall group (p < 0.001) BAI: inferior nasal wall group (p = 0.004); lateral nasal wall group (p < 0.001)	No complications reported
Lemogne et al. (2015)	Prospective case report	Pharmacological and cognitive therapy	1 (M)	Somatic disorder treatment, consisting of cognitive therapy to manage avoidance behaviour and dysfunctional beliefs, and Venlafaxine for symptomatic relief	18	Other: Clinicians’ observation and Subjective report by patient and family	Cognitive therapy and venlafaxine treatment resulted in functional improvements without relapse	No complications reported
Malik et al. (2021)	Prospective case-series	Implantation	5 (2M; 3F)	Half-dome-shaped cadaveric rib graft was implanted into a submucosal pocket. A 1–2 mm airway was maintained between the nasal septum and neo-turbinate (see Velasquez et al., 2015)	7.2 ± 1.4	Questionnaire: ENS6Q Clinical test: CFD	ENS6Q: 14 vs*.* 4.8 (6 months, p = 0.02) CFD evaluation showed an increase in airflow through inferior and superior meatuses and decrease in airflow through middle meatuses	N/R
McRoberts (2016)	Prospective case report	Neurostimulation	1 (M)	Implantation of two Octrode™ leads (peripheral V2 trigeminal lead and a C1-C2 lead) in cross-talk configuration to stimulate the trigeminocervical complex	36	Other: Subjective pain tolerance rating	Three years post-implantation patient experienced no baseline pain and was able to control exacerbations	N/R
Modrzyński (2011)	Prospective case-series	Injection	3 (2M; 1F)	Hyaluronic acid was injected into the inferior nasal concha and the submucosal membrane	12	Clinical test: Endoscopy, acoustic rhinometry	Endoscopic and acoustic rhinometry confirmed no depletion of the injection and a reduction in the nasal cavity volume	66.7% of patients experienced resorption of hyaluronic acid (12 months pot-op). Adverse events managed by reintervention (N = 1)
Rice (2000)	Prospective case report	Implantation	1 (F)	Hydroxyapatite cement was used to fill a subperiosteal pocket	12	Clinical test: CT-scan	CT-scan showed reduction in nasal cavity volume and stability of the implant	N/R
Saafan (2013)	Prospective RCT	Implantation	24 (11M; 13F)	Two treatment groups: 1. i) silastic group (n = 12): three submucosal pockets were implanted with 2 silastic strips each. ii) AlloDerm group (n = 12): three submucosal pockets were implanted with 5 AlloDerm sheets each	18 (9–24)	Questionnaire: SNOT-25 Clinical test: Endoscopy, anterior rhinoscopy	SNOT-25: silastic group 61.4 vs*.* 33.6 (p < 0.001); AlloDerm group 63.7 vs*.* 34.2 (p < 0.001) Endoscopy and anterior rhinoscopy rapid mucosa healing and improved nasal crusting	In AlloDerm group: 25% of patients experienced graft exposure (2w post-op); 16.6% of patients experienced graft volume loss (2 months post-op) In silastic group: 33.3% of patients experienced implant extrusion. Adverse events remained without any functional impact
Tam et al. (2014)	Retrospective case-series	Implantation	16 (10M; 6F)	Multiple pieces of Medpor® sheet were used to fill a submucosal pocket. The implant volume was based on patient anatomy	48	Questionnaire: SNOT-22 Clinical test: CT-scan, endoscopy	SNOT-22: 39.25 vs*.* 19.81 (3 months, p < 0.05), 16.19 (12 months, p < 0.05) CT-scan and endoscopy examinations showed reduction in nasal cavity volume	6.2% of patients experienced chronic hypertrophic rhinitis (48 months, post-op.) treated with nasal steroid spray 6.2% of patients experienced implant protrusion (6 months post-op) treated by partial implant removal
Thamboo et al. (2020)	Prospective case-series	Implantation	10 (7M; 3F)	Two treatment groups: 1. Small intestine submucosal (SIS) xenograft (Biodesign®) group (n = 3): two rolled SIS sheets were implanted submucosally. 2. AlloDerm group (n = 7): multiple AlloDerm pieces were placed within the submucosal pocket	6	Questionnaire: SNOT-22, ENS6Q, GAD-7, PHQ-9	SNOT-22: 62.82 vs*.* 54.4 (1w, p > 0.05), 43.88 (1 month, p > 0.05), 32.5 (3 months, p < 0.001), 31.63 (6 months, p < 0.001) ENS6Q: 21.55 vs*.* 14.14 (1w, p > 0.05), 10.89 (1 month, p > 0.05), 9.88 (3 months, p < 0.001), 8.5 (6 months, p < 0.001) GAD-7: 14.64 vs*.* 8 (1w, p > 0.05), 8.56 (1 month, p > 0.05), 4.75 (3 months, p < 0.01), 5.13 (6 months, p < 0.01) PHQ-9: 14.91 vs*.* 9.14 (1w, p > 0.05), 7.56 (1 month, p < 0.05), 4.88 (3 months, p < 0.001), 6.63 (6 months, p < 0.01)	10% of patients experienced graft resorption. Adverse events managed by replacing the small intestinal submucosal implant with AlloDerm
Tian et al. (2021)	Prospective case-series	Pharmacological and cognitive therapy	28 (13M; 15F)	Somatic disorder treatment consisting of cognitive behavioural therapy to manage dysfunctional beliefs, and serotonin reuptake inhibitors for depression and anxiety	12	Questionnaire: SNOT-25, GAD-7, PHQ-9, PHQ-15	SNOT-25: 64.89 vs*.* 46.5 (3 months, p < 0.001), 21.6 (12 months, p < 0.001) GAD-7: 10.54 vs*.* 1.54 (12 months, p < 0.001) PHQ-9: 10.64 vs*.* 1.96 (12 months, p < 0.0001) PHQ-15: 11.14 vs*.* 3.07 (12 months, p < 0.0001)	17.86% of patients experienced dry mouth; 10.71% of patients experienced dizziness; 7.14% of patients experienced sleeping disorder. All adverse events occurred within the first week of intervention and disappeared after 2 weeks without any treatment
Ushio et al. (2022)	Prospective case-series	Implantation	6 (5M; 1F)	Four to five pieces of autologous auricular cartilage were used to fill a submucosal pocket. Implantation site was guided by cotton test results	14.8 ± 4.8 (9–23)	Questionnaire: SNOT-20, SNOT-25, ENS6Q Clinical test: CT-scan, rhinomanometry	SNOT-20: 48.8 vs*.* 30.2 (p = 0.03) SNOT-25: 63.3 vs*.* 38.4 (p = 0.03) ENS6Q: 18 vs*.* 9.7 (p = 0.04) CT-scan showed nasal cavity volume reduction. Rhinomanometry showed an increase in nasal resistance (0.13 ± 0.03 vs*.* 0.16 ± 0.02 Pa/cm^3^/s)	No complications reported
Velasquez et al. (2015)	Prospective case-series	Implantation	3 (1M; 2F)	Four rectangular pieces of small intestine submucosal xenograft (Biodesign®) were rolled and implanted in a submucosal pocket. Additional small pieces were used to fill remaining gaps. while maintaining an airway space	3	Questionnaire: SNOT-25	SNOT-25: 77.6 vs*.* 65.3 (1w, N/R), 57 (1 month, p < 0.01), 55 (3 months, p < 0.01)	Mild partial reabsorption of the implant (12w, post-op)
Xu et al. (2015)	Prospective case-series	Injection	30 (20M; 10F)	Four nasal mucosal injections of autologous adipose-derived stem cells (ADSC) were conducted every 10 days. In patients without residual turbinate (n = 2) after three doses, ADSC mixed with autologous fat particles were submucosally injected	(9–18)	Clinical test: Endoscopy, acoustic rhinometry, histology, MCC Other: Patients’ subjective report	Endoscopy showed a more erythematous nasal cavity, increased mucus secretion and fewer scrabs. Acoustic rhinometry showed reduction in nasal cavity volume, improvement in nasal resistance and minimum cross-sectional area (9 months, p < 0.05). MCC improved post-op. (6 months, p < 0.05)	N/R

Summary of study design, interventions, patient numbers, follow-up, outcomes, results, and adverse events for each included study. Values of follow-up are presented as mean, range, mean ± SD, mean ± SD (range), mean (range), as made available by the authors. M, Male; F, Female; N/R, Not Reported; post-op, post-operative; post-int, post-intervention; w, week; mo, month; NOSE, Nasal Obstruction Symptom Evaluation; RhinoQoL, Rhinosinusitis Quality of Life questionnaire; ENS6Q, Empty Nose Syndrome 6-Item Questionnaire; PHQ-9/15, 9-item Patient Health Questionnaire; GAD-7, Generalized anxiety disorder; SNOT-20/22/25, Sino-Nasal Outcome Test; SS-12, 12-items odour identification test; BDI-II, Beck depression inventory II; BAI, Beck anxiety inventory; hs-CRP, high sensitivity-C reactive Protein; MCC, mucociliary clearance; PSQI, Pittsburgh Sleep Quality Index; EpSS, Epworth Sleepiness Scale; CFD, Computational Fluid Dynamics; VAS, Visual analogue scale.

### 3.2 Study characteristics and population

**FIGURE 2:**
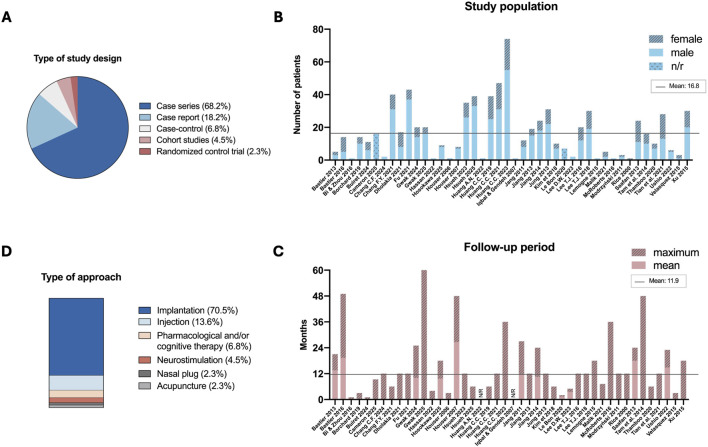
Overview of the included studies and patient populations. **(A)** Pie chart showing the distribution of studies based on study design. **(B)** Histograms representing the number of patients analyzed and sex distribution in each study. **(C)** Histograms showing the follow-up duration reporting, for the included studies, the maximum and mean values when available. **(D)** 100% stacked bar chart depicting the frequency of different treatment approaches for ENS. When ranges were reported, the mean value was used to calculate the overall mean. The horizontal black line indicates the mean of the respective parameters. N/R, not reported.

The majority of studies were designed as case series (30 studies, 68.2%), followed by case reports (8 studies, 18.2%), case-control studies (3 studies, 6.8%), cohort studies (2 studies, 4.5%), and one randomized controlled trial (Figure 2A). Among these, 79.5% (n = 35) were prospective studies and 20.5% (n = 9) retrospective studies.

Sample sizes varied across included studies, ranging from 1 to 74 individuals, with varying sex distribution. The average patient enrollment displayed relatively small cohort sizes, with a mean of 16.8 patients per study (Figure 2B). Notably, 32% of studies included five or fewer patients. Additionally, sex distribution data for treated patients, reported in 40 of the 44 studies included in the review, indicate a higher proportion of male patients (66.9%) than female patients (29.7%).

Study characteristics were analyzed and grouped into five macro-themes - type of approach, follow-up period, outcome measures, outcome measurements, and adverse events - and related micro-themes for implant-based approaches (material, dimension, N. of implants, location) or injection-based approaches (material, injection site, injected volume, N. of doses), allowing a structured data synthesis.

### 3.3 Follow-up period

The investigated articles exhibited a wide spectrum of follow-up durations, ranging from 1 to 61.2 months, with most studies having short-to-mid-term observation period. Indeed, 61.4% of reports had monitoring periods of less than 12 months, including eight studies lasting less than 6 months. A few studies stood out for their extensive observational periods, reaching up to 4 years (Bastier et al., 2016; Houser, 2007; Tam et al., 2014) or 5 years (Gwak and Jang, 2026) (Figure 2C).

### 3.4 Type of approach

The experimental treatments for ENS reported in the included studies demonstrated substantial diversity in therapeutic approaches, emphasizing the need for standardized intervention models. Implant-based treatments were the most prevalent intervention, featured in 31 studies (70.5%) (Figure 2D; Table 1). Six studies (13.6%) explored injection-based approaches (Modrzyński, 2011; Xu et al., 2015; Kim et al., 2018; Borchard et al., 2019; Lee et al., 2023; Buiret, 2024). Pharmacological and/or cognitive therapies were evaluated in three records (W Iqbal and Gendeh, 2007; Malik et al., 2021; Tian et al., 2021), while neurostimulation approaches were performed in two studies (McRoberts, 2016; Le Bon et al., 2020). Finally, one study tested a custom-made 3D-printed wearable nasal plug (Gwak et al., 2024), and another investigated acupuncture as a treatment to manage ENS (Bi and Zhou, 2016).

#### 3.4.1 Implant material

Within the implant-based approaches, 15 studies employed biologic materials (Table 2) (Houser, 2006; 2007; Jang et al., 2011; Jung et al., 2013; Saafan, 2013; Velasquez et al., 2015; Lee et al., 2016; Thamboo et al., 2020; Chang et al., 2021; Dholakia et al., 2021; Malik et al., 2021; Ushio et al., 2022; Cameron et al., 2025; Gwak and Jang, 2026; Hosokawa et al., 2025), while 18 studies used synthetic alternatives (Rice, 2000; Bastier et al., 2013; Bastier et al., 2016; Jiang et al., 2013; Jiang et al., 2014; Saafan, 2013; Tam et al., 2014; Lee et al., 2016; Lee et al., 2018; Huang et al., 2019; Huang et al., 2021; Huang et al., 2022a; Huang et al., 2023; Chang et al., 2021; Fu et al., 2021; Hassan et al., 2022; Hsueh et al., 2023; Hsueh et al., 2025).

**TABLE 2:** Study characteristics of implant and injection-based approaches.

Study	Material implanted	Implant site	Laterality	N° of implants/side	Graft dimensions cm/volume (ml/cm^3^)
Bastier et al. (2013)	ß -tricalcium phosphate	Lateral nasal wall	Bilateral	2	1.5 × 0.5 × 0.2
Bastier et al. (2016)	ß -tricalcium phosphate	Lateral nasal wall	Unilateral (n = 5); Bilateral (n = 9)	2	1.5 × 0.5 × 0.2
Cameron et al. (2025)	Purified bovine-derived collagen matrix	Remnant inferior turbinate (n = 1); remnant inferior turbinate and lateral nasal wall (n = 1); lateral nasal wall and nasal floor (n = 1); lateral nasal wall (n = 13)	Unilateral (n = 5); Bilateral (n = 11)	N/R	N/R
Chang (2024)	Platelet-rich fibrin scaffolds embedded with a diced cartilage graft	Lateral nasal wall	Unilateral	N/R	N/R
Chang et al. (2021)	Medpor®	Lateral nasal wall	N/R	N/R	0.8 × 2.5–0.8 × 4
Dholakia et al. (2021)	Cadaveric rib cartilage graft	Remnant inferior turbinate	Unilateral (n = 3); Bilateral (n = 14)	2	3 × 0.5 × 0.6
Fu et al. (2021)	Medpor®	Lateral nasal wall	N/R	N/R	0.8 × 2.5–0.8 × 4
Gwak and Jang, 2026	Autologous costal cartilage	Lateral nasal wall	Unilateral (n = 7); Bilateral (n = 13)	N/R	N/R
Hassan et al. (2022)	GlassBONE™	Lateral nasal wall and nasal floor	Bilateral	1	10 mL
Hosokawa et al. (2025)	Autologous dermal fat	Nasal floor	N/R	1	3 × 8
Houser (2006)	Acellular dermis (AlloDerm) and Cymetra (injectable Acellular dermis)	Nasal septum	Unilateral	2	1 × 2
Houser (2007)	Acellular dermis (AlloDerm)	Nasal septum and nasal floor (n = 1); septum, nasal floor and remnant inferior turbinate (n = 1); remnant inferior turbinate (n = 2); nasal septum (n = 3); remnant inferior turbinate and vestibular implantation (n = 1)	Unilateral (n = 5); Bilateral (n = 3)	N/R	N/R
Hsueh et al. (2023)	Medpor®	Nasal floor	N/R	N/R	0.8 × 2.5–4
Hsueh et al. (2025)	Medpor®	Nasal floor	N/R	N/R	0.8 × 2.5–4
Huang A.N. et al. (2022)	Gore-Tex®	Nasal floor	Unilateral	N/R	N/R
Huang et al. (2019)	Medpor®	Lateral nasal wall	N/R	N/R	0.8 × 2.5–0.8 × 4
Huang et al. (2021)	Medpor®	Lateral nasal wall	N/R	N/R	0.8 × 2.5–0.8 × 4
Huang et al. (2023)	Medpor®	N/R	Unilateral (n = 21); Bilateral (n = 53)	N/R	N/R
Jang et al. (2011)	Septal, conchal, autologous, or homologous costal cartilage	Lateral nasal wall	N/R	N/R	N/R
Jiang et al. (2013)	Medpor®	Lateral nasal wall; nasal floor and/or septum	Bilateral	1–4	0.3–1.3 x 2–3.8 × 0.3
Jiang et al. (2014)	Medpor®	Lateral nasal wall; nasal floor and/or septum	Bilateral	1–4	0.3–1.3 x 2–3.8 × 0.3
Jung et al. (2013)	Autologous conchal cartilage, Autologous/homologous costal cartilage	Lateral nasal wall	Unilateral (n = 2); Bilateral (n = 29)	N/R	2 cm^3^
Lee et al. (2016)	Medpor® or autologous septal bone	Lateral nasal wall	N/R	N/R	0.8 × 2.5–0.8 × 4
Lee et al. (2018)	Medpor®	Inferior or lateral nasal wall	N/R	N/R	0.8 × 2.5–4
Malik et al. (2021)	Cadaveric rib cartilage graft	Lateral nasal wall	N/R	N/R	3.5 × 0.7 × 0.6
Rice (2000)	Hydroxyapatite cement	Lateral nasal wall	Unilateral	N/R	N/R
Saafan (2013)	Silastic implant or Acellular dermis (AlloDerm)	Lateral nasal wall, nasal septum and nasal floor	Bilateral	6 silastic strips or 15 AlloDerm disks	N/R (Silastic); 1 × 0.5 × 0.1 (AlloDerm)
Tam et al. (2014)	Medpor®	nasal floor (n = 12) nasal floor and septum (n = 4)	Unilateral (n = 9); Bilateral (n = 7)	N/R	0.8 × 2.5–4
Thamboo et al. (2020)	Small intestine submucosal xenograft (Biodesign®) or Acellular dermis (AlloDerm)	Lateral nasal wall	Bilateral	2 (Biodesign®); ∼10 (AlloDerm)	2 × 3 (Biodesign®); 0.5 × 1 (AlloDerm)
Ushio et al. (2022)	Autologous auricular cartilage	Nasal floor	Unilateral (n = 1); Bilateral (n = 5)	4–5	N/R
Velasquez et al. (2015)	Small intestine submucosal xenograft (Biodesign®)	Lateral nasal wall	Bilateral	Unclear	Unclear

Study	Material injected	Injection site	Laterality	N° of injections	Volume injected (mL)
Borchard et al. (2019)	Carboxymethylcellulose gel (Prolaryn®)	Lateral nasal wall, remnant inferior turbinate, nasal vestibuleetc.	Unilateral (n = 3); Bilateral (n = 11)	4–5	0.4–0.8
Buiret (2024)	Autologous adipocytes	Remnant inferior turbinate, lateral nasal wall, nasal floor	Unilateral (n = 5); Bilateral (n = 6)	3	4.5–8
Kim et al. (2018)	Autologous fat-derived stromal vascular fraction	Remnant inferior turbinate	Bilateral	1	1.5
Lee et al. (2023)	Autologous platelet-rich plasma	Remnant inferior turbinate	Bilateral	10–12	N/R
Modrzyński (2011)	Hyaluronic acid	Remnant inferior turbinate, nasal septum	Bilateral	2	∼0.3–0.4
Xu et al. (2015)	Autologous adipose-derived stem cells (ADSCs) w/wo fat particles	Area of nasal mucosa damage	Bilateral	4	1–5 × 10^7^ ADSCs w/wo 1 × −5 (autologous fat)

Overview of implanted/injected materials, intervention sites, laterality, number of implants/injections, and graft size or volume. N/R, not reported; w/wo, with or without.

**FIGURE 3:**
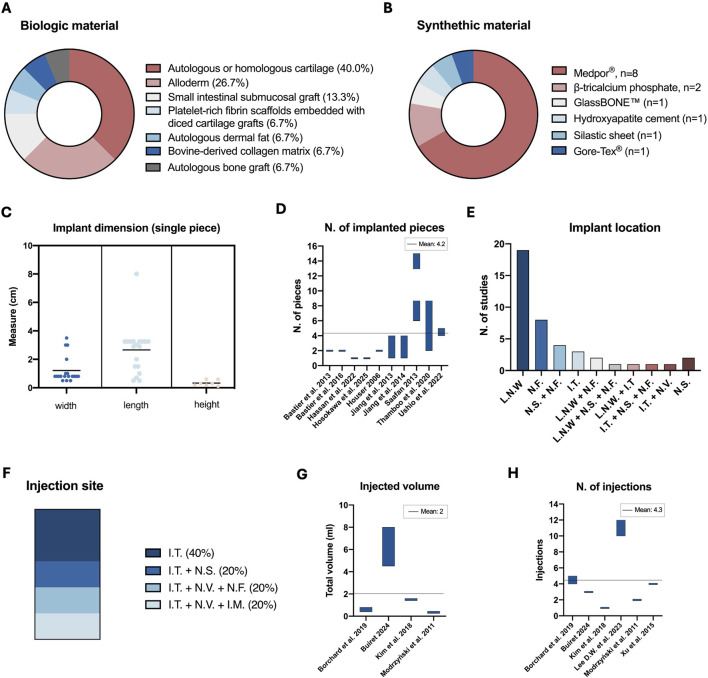
Summary of materials used and characteristics of implants and injections treatments. **(A)** Pie chart showing the types and frequency of biological implants used. **(B)** Pie chart showing the types and frequency of synthetic implants used. **(C)** Scatter plot illustrating the width, length, and height of individual implanted material pieces; for ranges, the mean value is reported. **(D)** Floating bar chart illustrating the range or number of implanted pieces reported for each study. **(E)** Histograms depicting implant locations and the number of studies in which each location was utilized. **(F)** Stacked bar chart showing the site and relative frequency of injection locations. **(G)** Floating bar chart reporting the range or volume of a single injection. **(H)** Floating bar chart showing the range or number of injections performed in each study. The horizontal black line indicates the mean of the respective parameters. L.N.W., Lateral nasal wall; N.F., Nasal floor; N.S., Nasal septum; I.T., Inferior turbinate; N.V.; Nasal vestibule; I.M., Inferior meatus.

Among the biological options (Figure 3A), cartilage tissue emerged as the most frequently used material, appearing in seven studies. Five groups used autologous or homologous costal cartilage (Jang et al., 2011; Jung et al., 2013; Dholakia et al., 2021; Malik et al., 2021; Gwak and Jang, 2026), including two that implanted cadaveric rib grafts (Dholakia et al., 2021; Malik et al., 2021) and two that also employed nasal cartilage (septal or conchal). Auricular cartilage was implanted in one study (Ushio et al., 2022), while Chang C.F. and colleagues (Chang et al., 2021) introduced a combined technique using diced cartilage integrated with platelet-rich fibrin scaffolds.

AlloDerm™, an acellular dermal matrix derived from human skin, was investigated in four studies (Houser, 2006; Houser, 2007; Saafan, 2013; Thamboo et al., 2020). In Houser (2006), the AlloDerm™ implant was followed by the placement of an injectable acellular dermis (Cymetra). Other autologous approaches included turbinate augmentation with dermal fat (Hosokawa et al., 2025) and septal bone graft implantation (Lee et al., 2016). Non-autologous approaches included porcine small intestinal submucosal implants (Velasquez et al., 2015; Thamboo et al., 2020) and bovine-derived collagen matrix (Cameron et al., 2025).

Among the synthetic implants (Figure 3B; Table 2), Medpor®, a biocompatible porous polyethylene material, was by far the most widely used (Jiang et al., 2013; Saafan, 2013; Jiang et al., 2014; Lee et al., 2016; Lee et al., 2018; Huang et al., 2019; Chang et al., 2021; Fu et al., 2021; Huang et al., 2021; Hsueh et al., 2023; Huang et al., 2023; Hsueh et al., 2025). Other materials included β-tricalcium phosphate (Bastier et al., 2013; Bastier et al., 2016), GlassBONE™ (Hassan et al., 2022), hydroxyapatite cement (Rice, 2000), silastic sheets (Saafan, 2013) and expanded polytetrafluoroethylene (Gore-Tex®) (Huang A. N. et al., 2022).

In most implant-based studies, the surgical approach involved an incision at the selected recipient site followed by elevation of a submucoperiosteal flap to form a submucosal pocket for placement of the chosen material (Table 1). The implant was then secured by closing the pocket using resorbable sutures, fibrin sealant, or Hemopatch®.

#### 3.4.2 Implant dimension

In the majority of the implant-based studies, researchers reported inserting rectangular pieces cut or reshaped from the original material, stacked or arranged side by side depending on the length of the patient’s nasal floor or inferior meatus (Table 2). The average width of each piece was 1.21 ± 0.93 cm, and the mean length was 2.66 ± 1.68 cm. Only a few studies (Bastier et al., 2013; Bastier et al., 2016; Jiang et al., 2013; Jiang et al., 2014; Saafan, 2013; Dholakia et al., 2021; Malik et al., 2021) reported the height of each piece, with an average of 0.33 ± 0.20 cm (Figure 3C). Jung and colleagues reported implanting round pieces of autologous or homologous cartilage measuring 2 cm^3^ (Jung et al., 2013), while another study reported bilateral submucoperiosteal implantation using 10 mL of a synthetic ceramic, GlassBONE™ (Hassan et al., 2022). The number of rectangular pieces reported ranged from 1 to 15, with a mean of 4.2 (Figure 3D). Finally, eleven studies did not detail the precise dimensions of the implant (Rice, 2000; Houser, 2007; Jang et al., 2011; Velasquez et al., 2015; Chang et al., 2021; Huang et al., 2021; Huang A. N. et al., 2022; Huang et al., 2023; Ushio et al., 2022; Cameron et al., 2025; Gwak and Jang, 2026).

#### 3.4.3 Implant location

Across the included studies, the most common implantation site was the lateral nasal wall, serving as the sole location in 19 studies (Houser, 2006; Jang et al., 2011; Bastier et al., 2013; Jiang et al., 2013; Jung et al., 2013; Jiang et al., 2014; Velasquez et al., 2015; Bastier et al., 2016; Lee et al., 2016; 2018; Huang et al., 2019; Chang et al., 2021; Fu et al., 2021; Malik et al., 2021; Tian et al., 2021; Cameron et al., 2025; Gwak and Jang, 2026; Rice, 2000). The nasal floor was targeted in 8 studies (Jiang et al., 2013; Jiang et al., 2014; Tam et al., 2014; Huang A. N. et al., 2022; Ushio et al., 2022; Hosokawa et al., 2025; Hsueh et al., 2025) (Figure 3E; Table 2). Two studies reported simultaneous graft placement in the lateral nasal wall and nasal floor (Hassan et al., 2022; Cameron et al., 2025), while five studies opted for the nasal septum alone (Houser, 2006; 2007) or in combination with the nasal floor (Houser, 2007; Jiang et al., 2013; Jiang et al., 2014; Tam et al., 2014) (Figure 3E). The remnant inferior turbinate was also targeted as an implantation site, either independently (Houser, 2007; Dholakia et al., 2021; Cameron et al., 2025) or combined with the lateral nasal wall (Cameron et al., 2025), vestibular region (Houser, 2007), nasal septum, and nasal floor (Houser, 2007). One study performed simultaneous implantations in lateral nasal wall, nasal septum and nasal floor (Saafan, 2013), while another compared lateral and inferior nasal wall implantation across two experimental groups (Lee et al., 2018) (Figure 3E).

#### 3.4.4 Injection-based approach: injected material, injection site, injected volume, N. of injections

Among the six studies investigating augmentation through injection (Table 2), three employed cell-based or cell-derivatives autologous approaches, including adipocytes harvested via umbilical puncture (Buiret, 2024), fat-derived stromal vascular fraction (Kim et al., 2018), and adipose-derived stem cells with or without autologous fat particles (Xu et al., 2015). One study used autologous platelet-rich plasma (Lee et al., 2023). In contrast, acellular injectable fillers consisted of commercially available materials such as Prolaryn® (carboxymethylcellulose with glycerin gel) (Borchard et al., 2019) and hyaluronic acid (Modrzyński, 2011). Most cell-based techniques involved minimal manipulation, except for Xu and colleagues, who cultured fat-derived cells for three passages before injection (Xu et al., 2015). Additional manipulation included preparation of a high-density stromal vascular fraction pellet from adipose tissue (Kim et al., 2018).

Across the included studies, the remnant inferior turbinates were the most frequently targeted sites, with injections directed toward their head, body, or medial surface (Modrzyński, 2011; Kim et al., 2018; Borchard et al., 2019; Lee et al., 2023; Buiret, 2024) (Figure 3F). Some protocols performed initial injections at the inferior turbinate followed by additional sites such as the lateral wall of the nasal valve, and anterior nasal cavity floor (Buiret, 2024), while others targeted also the lateral nasal wall and the anterior nasal vestibule (Borchard et al., 2019), or the submucosal septal regions (Modrzyński, 2011) (Figure 3F; Table 2). Finally, Xu et al. reported injections performed in areas of nasal mucosal damage (Xu et al., 2015).

Concerning treatment laterality, four studies performed bilateral injection (Modrzyński, 2011; Xu et al., 2015; Kim et al., 2018; Lee et al., 2023), whereas two studies included both unilateral and bilateral procedures (Borchard et al., 2019; Buiret, 2024). When reported, injected volumes ranged from 0.35 to 5.5 mL per side, with an average of 2.0 mL (Figure 3G; Table 2).

Operatively, injections were administered either during a single session with 1-5 injections performed (Modrzyński, 2011; Kim et al., 2018; Borchard et al., 2019; Buiret, 2024), or across multiple sessions, such as 10–12 injections over two to three months (Lee et al., 2023), or one injection every 10 days for four sessions (Xu et al., 2015) (Figure 3H; Table 2).

#### 3.4.5 Alternate treatment strategies

Among studies that explored alternatives to implantation or injection, two investigated neurostimulation-based approaches for ENS management. Specifically, one utilized a non-invasive method involving intranasal trigeminal training with levomenthol and eucalyptol inhalations administered three times daily for at least 30 days to stimulate the Transient Receptor Potential 8 (TRPM8) (Le Bon et al., 2020). The other study applied a neuromodulation technique by implanting trigeminal and C1–C2 leads to stimulate the trigeminocervical complex (McRoberts, 2016). Three studies adopted pharmacological or combined therapeutic approaches. One focused on symptomatic relief using topical and systemic medications, including steroid nasal sprays, mucolytics, oral antibiotics, saline preparations, and a leukotriene receptor antagonist (W Iqbal and Gendeh, 2007). Instead, two studies integrated pharmacological treatment with cognitive therapy, using venlafaxine to manage symptoms (Lemogne et al., 2015) or selective serotonin reuptake inhibitors for depression and anxiety (Tian et al., 2021), alongside therapy targeting avoidance behavior and dysfunctional beliefs (Lemogne et al., 2015) or maladaptive thought patterns (Tian et al., 2021). Recently, the use of 3D-printed external prostheses has also been explored for ENS management. In particular, a wearable nasal plug composed of polyurethane elastomer with a shape-memory polymer loop was customized to fit patient-specific nasal cavities, with the head portion filling the defect and connecting turbinate and lateral wall mucosa (Gwak et al., 2024). Finally, acupuncture needles inserted at multiple pressure points, with either reinforced and/or reduced manipulation, have been tested (Bi and Zhou, 2016).

### 3.5 Outcomes measures

**FIGURE 4:**
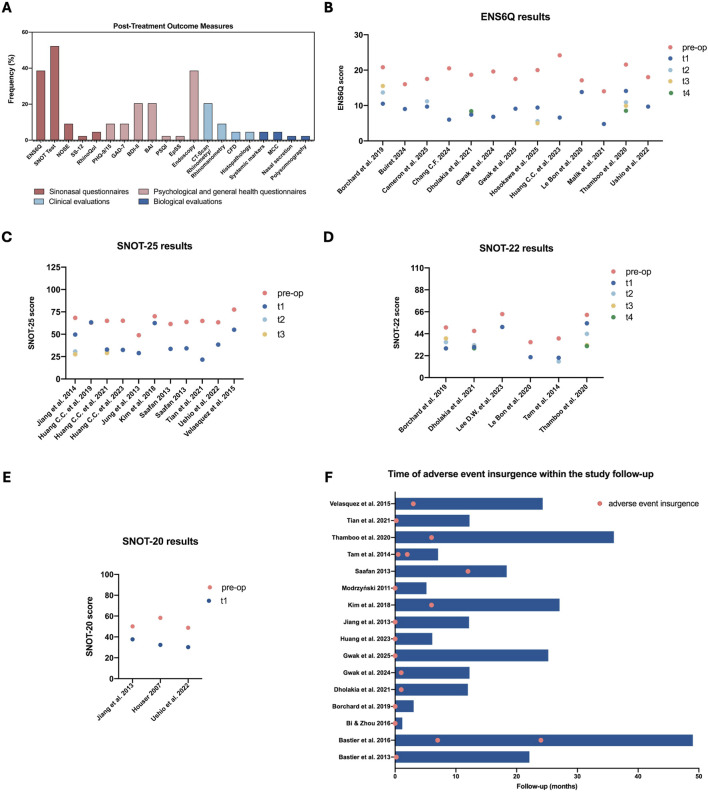
Post-treatment outcomes and adverse events analysis. **(A)** Usage frequency of the various post-treatment outcome measures across the included studies. **(B)** ENS6Q score values at pre-operative baseline and progressive follow-up time points (t1–t4). **(C)** SNOT-25 score values at pre-operative baseline and progressive follow-up time points (t1–t3). **(D)** SNOT-22 score values at pre-operative baseline and progressive follow-up time points (t1–t4). **(E)** SNOT-20 score values at pre-operative baseline and follow-up time point (t1). **(F)** Occurrence of different adverse events (red dots) along the total duration of follow-up, indicated by blue bars. The early onset of adverse events suggests that the tested interventions could not be optimized for the ENS-specific microenvironment. ENS6Q, Empty Nose Syndrome 6-Item Questionnaire; SNOT-20/22/25, Sino-Nasal Outcome Test; NOSE, Nasal Obstruction Symptom Evaluation; SS-12, 12-items odour identification test; RhinoQoL, Rhinosinusitis Quality of Life questionnaire; PHQ-9/15, 9-item Patient Health Questionnaire; GAD-7, Generalized anxiety disorder; BDI-II, Beck depression inventory II; BAI, Beck anxiety inventory; PSQI, Pittsburgh Sleep Quality Index; EpSS, Epworth Sleepiness Scale; CFD, Computational Fluid Dynamics; MCC, mucociliary clearance; pre-op, pre-operative; t, time-point.

Outcome measurements during follow-up were primarily via questionnaires, used in 33 of 44 studies (75%), occasionally alongside other assessments. Clinical evaluations were conducted in 22 of the included studies (50%), while only 6 studies (13.6%) assessed biological parameters, and 4 studies (9.1%) relied solely on non-validated subjective patient reports (Table 1). The SNOT questionnaires were the most commonly employed, appearing in 23 studies (SNOT-25 in n = 13, SNOT-22 in n = 7, and SNOT-20 in n = 3). ENS6Q and the NOSE scores were applied in 17 and 4 studies, respectively (Figure 4A). Other sinonasal-specific questionnaires included the Rhino Quality of Life Questionnaire (RhinoQoL, 2 studies), and the 12-item Sniffin’ Sticks Odor Identification Test (SS-12, 1 study). Several studies also incorporated psychological self-report measures, such as the Beck Depression Inventory-II (BDI-II) and the Beck Anxiety Inventory (BAI) (9 studies), as well as the Generalized Anxiety Disorder-7 (GAD-7) and the Patient Health Questionnaire (PHQ-9 or PHQ-15) (4 studies). Additionally, sleep quality was evaluated in one study using the Pittsburgh Sleep Quality Index (PSQI) and the Epworth Sleepiness Scale (EpSS).

Clinical evaluations commonly used nasal endoscopy (17 studies, 38.6%) and CT scans (9 studies, 20.4%) for patient follow-up, succeeded by acoustic rhinometry (4 studies, 9.1%), CFD and polysomnography (2 studies, 4.5%). Biological assessments included histological analysis (Houser, 2007; Xu et al., 2015), mucociliary clearance testing (Jiang et al., 2013; Xu et al., 2015), analysis of systemic markers (Huang et al., 2019; Fu et al., 2021), and nasal secretion analysis (Kim et al., 2018) (Figure 4A). Additionally, one study used the Visual Analogue Scale (VAS) to rate the severity level of five nasal and facial symptoms.

### 3.6 Outcome measurements

Overall, sinonasal-specific questionnaires (ENS6Q, SNOT, NOSE, RhinoQoL, SS-12) showed statistically significant improvements in 81.6% of comparisons between pre-operative values and latest post-treatment outcomes (Table 1). A comparable trend was observed for the psychological and general health questionnaires with statistically significant post-treatment improvement of 86.7%. When evaluating repeated follow-up durations ranging from 1 week to 1 year, seven studies reported a reduction in significance over time, nine showed stable results, and seven demonstrated higher significance at later follow-up, based on comparisons between preoperative and postoperative questionnaire scores (Figures 4B–E; Table 1). In the randomized controlled trial comparing implantation of synthetic silastic sheets with AlloDerm™, post-treatment SNOT-25 scores were comparable between groups, with both showing significant improvement **(**p < 0.001**)** (Saafan, 2013). When evaluating different implant locations, the lateral nasal wall group demonstrated significantly better postoperative SNOT-22 scores than the inferior nasal wall group **(**p < 0.001 vs. p = 0.002) (Lee et al., 2018). This advantage was corroborated by psychological outcome measures, with greater post-treatment improvement in depression and anxiety scores among patients with lateral wall implants (BDI-II: p < 0.001 vs. p < 0.05; BAI: p < 0.001 vs. p < 0.01) (Lee et al., 2018).

Regarding outcomes derived from post-treatment clinical assessments, nasal endoscopy and CT evaluations generally demonstrated effective narrowing of the nasal cavity, stable implant/injection position, and absence of volume loss (Rice, 2000; Bastier et al., 2013; Jiang et al., 2013; Jung et al., 2013; Tam et al., 2014; Hassan et al., 2022; Ushio et al., 2022; Lee et al., 2023; Chang, 2024; Cameron et al., 2025; Hosokawa et al., 2025). Injected hyaluronic acid remained stable up to 6 months but was fully resorbed after nine to twelve months (Modrzyński, 2011). Additional findings included absence of infection, rejection, or allergic reaction (Jung et al., 2013; Saafan, 2013), good mucosal healing (Jung et al., 2013; Hassan et al., 2022), smooth and intact mucosa covering the graft (Lee et al., 2016), improvement in nasal crusting (Saafan, 2013; Xu et al., 2015; Hassan et al., 2022), increased mucus secretion (Xu et al., 2015; Lee et al., 2016), resolution of mucosal dryness (Lee et al., 2023), and no evidence of nasal polyps or sinus pathology (Bastier et al., 2013). Acoustic rhinometry and rhinomanometry were assessed in four studies (Modrzyński, 2011; Jiang et al., 2013; Xu et al., 2015; Ushio et al., 2022) and revealed variable postoperative outcomes. Some studies observed gradual improvements in nasal resistance, nasal volume, and minimum cross-sectional area, reaching statistical significance at nine to twelve months (Jiang et al., 2013; Xu et al., 2015), while others observed early increases (three months) in nasal resistance (Ushio et al., 2022), or no significant nasal volume change (Modrzyński, 2011). CFD analyses demonstrated postoperative recovery of physiological airflow patterns and improved nasal conditioning (Malik et al., 2021; Huang A. N. et al., 2022). Huang found that surgery enhanced nasal air conditioning by redistributing airflow and increasing heat and water vapor fluxes, raising air temperature (Huang A. N. et al., 2022). Similarly, Malik reported restoration of airflow toward the inferior meatus after inferior meatus augmentation procedure, with air following the implant’s curved surface, suggesting restoration of native turbinate flow dynamics (Malik et al., 2021). Ultimately, the only study evaluating sleep through clinical examination found no statistically significant changes in polysomnographic parameters after treatment (Hsueh et al., 2025). Biological assessments via histology showed integrated implant with vascularization (Houser, 2007) and reduced lymphocyte-neutrophil infiltration with improved collagen organization (Xu et al., 2015). In studies evaluating mucociliary function, saccharin clearance times transiently improved between 3 and 6 months postoperatively but was not sustained at 9–12 months, indicating a non-significant enhancement (Jiang et al., 2013; Xu et al., 2015). Interestingly, high-sensitivity C-reactive protein analysis showed a significant 1-year postoperative decrease in peripheral blood levels among ENS patients with depression and anxiety, whereas no such change was observed in patients without those psychological conditions (Fu et al., 2021). In contrast, IgE serum levels showed no significant differences between pre-treatment and 6-month postoperative measurements (Huang et al., 2019). Concurrently, analysis of nasal secretions showed significantly decreased levels of the inflammatory cytokines IL-1β and IL-8, while non-significant reductions were observed in calcitonin gene-related peptide (CGRP) and lactoferrin (LTF) (Kim et al., 2018). Lastly, subjective outcomes were predominantly evaluated through patient-reported measures, including overall symptom reports (Xu et al., 2015; Bi and Zhou, 2016; Huang A. N. et al., 2022; Gwak et al., 2024), olfaction rating (Chang et al., 2021), and pain scoring (McRoberts, 2016), while Lemogne’s study appeared to rely on subjective reports from patients’ relatives (Lemogne et al., 2015).

### 3.7 Adverse events

Adverse events analysis was described in 64% of studies (28/44) (Table 1). Most reported undesirable effects were related to implant or injected material extrusion (Bastier et al., 2013; Jiang et al., 2013; Saafan, 2013), exposure into the nasal cavity lumen (Saafan, 2013; Bastier et al., 2016; Huang et al., 2023), protrusion (Tam et al., 2014; Bastier et al., 2016), reabsorption (Modrzyński, 2011; Velasquez et al., 2015; Thamboo et al., 2020), infection (Huang et al., 2023) or graft volume loss (Saafan, 2013). Instead, the use of external prosthesis was accompanied by discomfort due to nasal plug displacement (Gwak et al., 2024).

Surgery-related complications were described in four studies and included mild crusting, edema, nasal synechiae and epistaxis at the incision site (Dholakia et al., 2021), flap laceration and costal wound complications (Gwak and Jang, 2026)**,** temporary pressure sensation of the upper alveolar dentition (Borchard et al., 2019), and seroma formation (Kim et al., 2018). In addition, several postoperative symptoms reported by patients included headache (Bi and Zhou, 2016; Dholakia et al., 2021; Gwak and Jang, 2026)**,** foreign body sensation or decreased nasal comfort (Bastier et al., 2016; Gwak et al., 2024; Gwak and Jang, 2026), postnasal drip and nasal pain (Gwak et al., 2024; Gwak and Jang, 2026), nasal congestion and recurrent epistaxis (Gwak and Jang, 2026), itchiness and cosmetic concerns (Gwak et al., 2024), dizziness (Tian et al., 2021), chronic hypertrophic rhinitis (Tam et al., 2014), dry mouth and sleep disorders (Tian et al., 2021), neck stiffness, emesis, and eye swelling (Dholakia et al., 2021).

Notably, 42.9% of studies (12/28) that assessed adverse events reported no complications following treatment (Table 1). Among the studies that documented the timing of adverse event onset, most complications occurred within the first month (W Iqbal and Gendeh, 2007; Bastier et al., 2013; Saafan, 2013; Bi and Zhou, 2016; Dholakia et al., 2021; Tian et al., 2021; Gwak et al., 2024), with three studies recording events immediately after treatment (Bi and Zhou, 2016; Borchard et al., 2019; Dholakia et al., 2021). Beyond this period, three studies reported undesirable effects between two and three months (Saafan, 2013; Velasquez et al., 2015; Bi and Zhou, 2016), four studies between six and twelve months (Jiang et al., 2013; Tam et al., 2014; Bastier et al., 2016), one at two years (Bastier et al., 2016) and one at four years (Tam et al., 2014) (Figure 4F).

Management strategies primarily reported implant-related complications and included graft removal (Cameron et al., 2025; Gwak and Jang, 2026) or reduction (Tam et al., 2014; Gwak and Jang, 2026), typically undertaken in cases of nasal obstruction, implant protrusion, or insufficient patient-reported improvement. Additionally, in some cases, implantation was repeated due to resorption, misplacement, or extrusion (Jiang et al., 2013; Bastier et al., 2016; Thamboo et al., 2020; Huang et al., 2023); this included both standard replacement with a larger implant (Bastier et al., 2016) or with a different material (Thamboo et al., 2020).

### 3.8 Risk of bias

**FIGURE 5:**
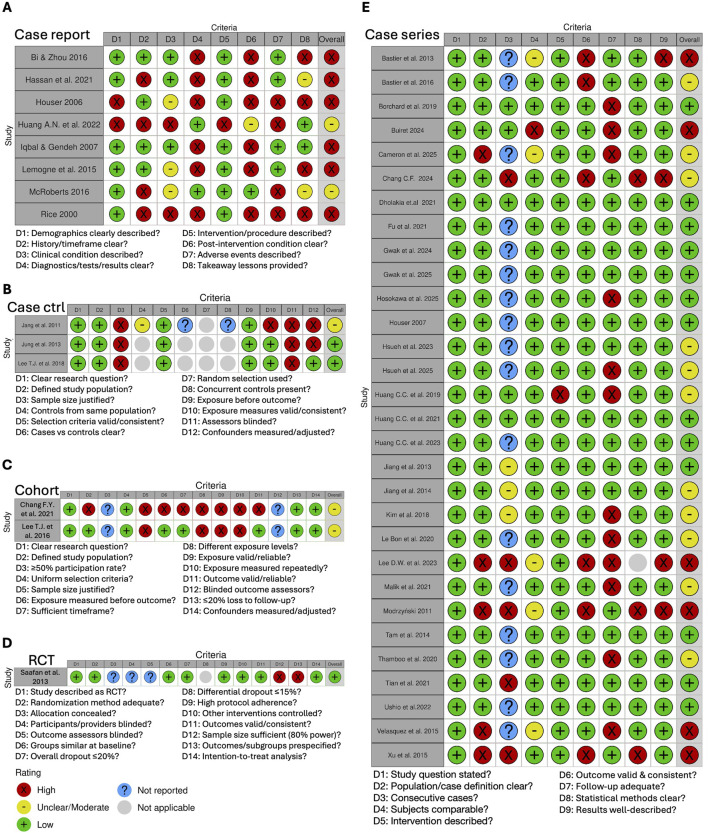
Risk-of-bias assessment across study designs. Results of the risk-of-bias evaluation conducted for **(A)** case reports, **(B)** case–control studies, **(C)** cohort studies, **(D)** randomized controlled trials, and **(E)** case series studies. RCT, randomized controlled trial.

**FIGURE 6:**
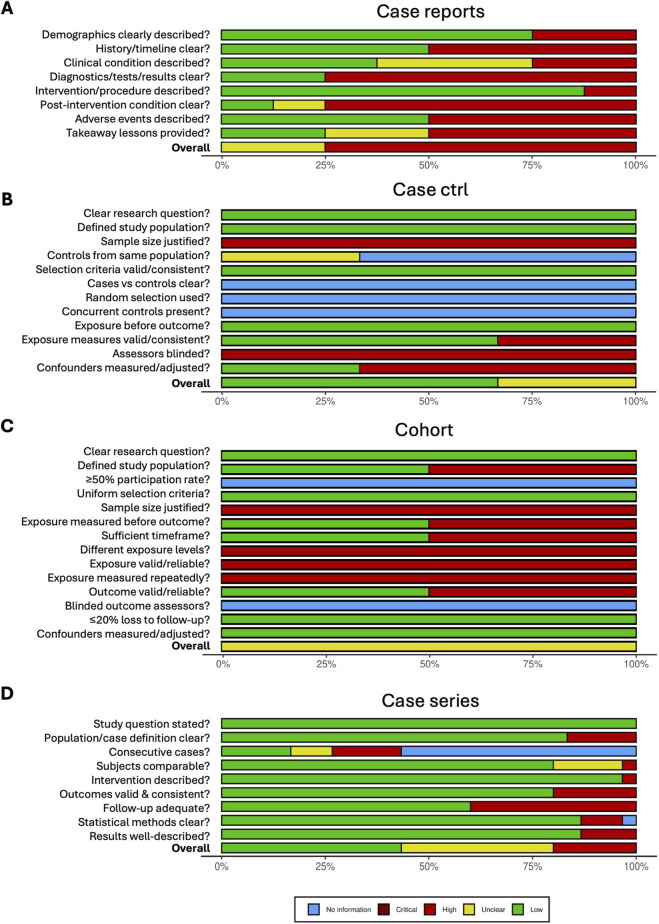
Summary plot of risk-of-bias assessment across study designs. Summary of the risk-of-bias evaluation performed for **(A)** case reports, **(B)** case–control studies, **(C)** cohort studies, and **(D)** case series studies.

The risk of bias assessment revealed variable methodological quality among the included studies. The highest risk of bias was observed in case report studies, with 6 out of 8 studies (75%) presenting a high risk, mainly due to poor reliability of diagnostic tests or assessment methods, as well as unclear reporting of results and post-intervention clinical condition (Figures 5A, 6A). Overall judgement of the case-control studies included in the review was rated as low-to-moderate risk of bias (Figure 6B), although their study design only partially met the conventional case-control framework. Specifically, these studies compared two reconstructive techniques instead of using true control groups. Therefore, potential placebo effects were not investigated. Consequently, domains related to the identification and selection of controls, comparability between cases and controls, and the use of concurrent controls were not applicable. The main sources of bias included the absence of sample size justification and the lack of blinding of assessors to case or control status (Figures 5B, 6B). Both cohort studies showed specific weaknesses in sample size justification and exposure assessment domains, resulting in an overall unclear to moderate risk of bias. (Figures 5C, 6C). The single randomized controlled trial received an overall positive risk of bias assessment (Figure 5D), although some concerns were noted regarding sample size and blinding of outcome assessment. Finally, among case series, 43.3% (13/30) were rated as low risk of bias, 36.7% (11/30) as moderate/unclear risk and 20% (6/30) as high risk (Figures 5E, 6D). The most problematic domains were insufficient data on the consecutiveness of patients included and follow-up duration shorter than one year, which limits robust outcome assessment. Additionally, outcome measures were not always clearly defined, validated, reliable, or consistently applied across participants.

## 4 Discussion

The broad range of therapeutic options described in the ENS treatment literature underscores the multifaceted nature of this syndrome for which surgical, regenerative, neurostimulation, and pharmacological-cognitive modalities have been explored. Despite efforts, none of the existing treatments has yet achieved the level of standardization and evidence-based proven efficacy required for routine clinical implementation. The critical assessment of the included studies helped to point out meaningful observations and limits of the current field of research on ENS. In the first instance, the predominance of observational and small-scale studies, with a limited number of case-control or randomized controlled trials, combined with uneven sex representation, short follow-up periods, and heterogeneity of outcome measures, hinders the generalizability of findings, limiting the evaluation of the long-term durability of treatments. Together, the current scenario underscores the need for rigorous, well-designed studies and supports the integration of diverse clinical experiences. In line with this, the risk of bias analysis suggests that future studies should more clearly report the involved population - clarifying whether patients were enrolled consecutively, applying well-defined selection criteria, and ensuring assessor blinding in case-control designs - to improve data reliability. Since sham surgery is ethically unacceptable for most proposed treatments and double-blind administration is often not feasible, potential solutions would be to use the natural progression of the pathology as a control group and the implementation of independent assessors to evaluate treatment outcomes objectively. However, defining objective evaluation metrics that can be consistently applied across different studies is, in itself, a major challenge that must be urgently addressed to guarantee the comparability, reproducibility, and clinical relevance of results. As pointed out from the literature analysis, to date, the efficacy of experimental ENS treatments has largely relied on patient-reported questionnaires and general clinical evaluations. In our view, this overdependence on qualitative outcomes, while important for assessing quality of life, reflects the limited understanding of ENS pathophysiology and should be complemented by less subjective measures. The incomplete characterization of the causal mechanisms driving ENS, including the diverse patterns of turbinate tissue damage and neuronal impairment, also hampers accurate diagnosis and patient stratification. Indeed, alongside identifying solutions to assess treatment efficacy, it is crucial to identify potential ENS patient subgroups based on the underlying injury type driving symptoms. Such stratification would guide the selection of the most appropriate experimental therapy for each group and enable the definition of objective, subgroup-specific outcome parameters. With the aim of classifying patients based on specific characteristics, a few biomarkers have been proposed to identify ENS patients at higher risk of psychiatric complications. Elevated preoperative levels of IL-6 and hs-CRP have respectively been associated with an increased incidence of suicidal thoughts and more severe depression (Hsueh et al., 2023), as well as persistent depressive symptoms one year after surgery (Fu et al., 2021). This evidence highlights the importance of timely interventions to prevent suffering, alongside close postoperative monitoring and psychotherapy. Moreover, airway biomarkers such as nasal nitric oxide have been linked to improvements in BDI-II and BAI scores, suggesting their role in monitoring psychiatric recovery (Fu et al., 2019). These findings collectively support the potential utility of inflammatory markers not only in risk stratification but also in tailoring postoperative management strategies. Furthermore, preoperative psychological factors - especially elevated BDI-II and BAI scores - along with female sex, may help guide patient selection for surgical intervention (Lee et al., 2016) and have predictive value for post-treatment residual symptoms (Lee et al., 2016; Huang et al., 2023; Huang et al., 2024). Histopathological analysis provides complementary insights that are useful for a deeper understanding of the complex and heterogeneous ENS pathophysiology, as well as for enabling a more thorough evaluation of treatment outcomes. Although applied in only a few of the included studies, biological assessments - such as histological analysis, mucociliary clearance, and secretion evaluation - have proven valuable in elucidating implant engraftment and mucosal functionality. Future ENS treatment studies should more systematically leverage characteristic histopathological features, such as airway remodeling with squamous and goblet cell metaplasia, submucosal fibrosis, reduced gland numbers, and decreased TRPM8 expression (Fu et al., 2021; Wu et al., 2021). In a comparison between the currently available reconstructive strategies for ENS management, Medpor®, cartilage grafts, and AlloDerm™ showed appreciable differences in efficacy, psychological outcomes, biological performance, and post-treatment complications. In terms of alleviating sino-nasal symptoms, all three materials led to significant relief, although the evidence was based on different follow-up durations. Psychological outcomes were more variable: Medpor® and cartilage grafts significantly reduced BAI and BDI-II scores but did not impact on GAD-7 or PHQ-9 scores, whereas AlloDerm™ also led to significant improvement in these latter two scores (Thamboo et al., 2020). From a surgical perspective, all three materials successfully fulfilled their primary purpose, reducing the nasal cavity volume. Medpor® allowed greater customization to suit the patient’s defect and showed no signs of implant infection, rejection, or allergic reaction, although evidence regarding the extent of mucosal recovery was limited to reduced crust formation (Saafan, 2013). In contrast, AlloDerm™ facilitated rapid mucosal healing and improved nasal crusting, but it was more susceptible to volume loss due to shrinkage and resorption (Houser, 2007; Saafan, 2013; Thamboo et al., 2020). The use of cartilage grafts, instead, not only provided stable implantation with enhanced nasal aerodynamics (Malik et al., 2021) and low infection and resorption rates but also promoted effective mucosal healing (Jung et al., 2013), with minimal complications mostly related to graft availability and donor site morbidity (Dholakia et al., 2021; Gwak and Jang, 2026). To overcome donor site morbidity and generate larger grafts from minimal invasive biopsies, tissue engineering can be used as a possible solution for ENS treatment, in line with successful clinical applications of regenerative medicine for the repair of nasal and articular defects (Fulco et al., 2014; Mumme et al., 2016; Kaiser et al., 2024). Evaluating the efficacy of injectable therapeutic approaches is even more challenging, as the limitations already observed in implant-based strategies are further amplified, including the very short follow-up periods that often reveal a rapid decline in effects, and an excessive reliance on heterogeneous and largely subjective outcome measures, which exhibit considerable variability. Additionally, adverse event reporting is infrequent and often inadequate, further limiting the safety and comparability of study results. Preliminary evidence suggests that autologous cell-based or cell-derivative treatments, including adipocytes, adipose-derived stem cells, stromal vascular fraction, and platelet-rich plasma, may provide some symptomatic relief, mainly through immunomodulatory mechanisms (Xu et al., 2015; Kim et al., 2018; Buiret, 2024), whereas acellular injectable fillers appear to produce even more transient or inconsistent effects (Modrzyński, 2011; Borchard et al., 2019).

Earlier literature reviews on ENS management have underscored that effective treatment strategies should target multiple aspects of the disease to maximise patients’ health (Gordiienko et al., 2021; Hussain et al., 2024). Specifically, three main challenges have been identified, namely: i) to recover proper nasal aerodynamics by reducing nasal cavity volume, ii) to promote healing of the damaged nasal mucosa and submucosal tissue, and iii) to stimulate nerve regeneration and restore normal tissue sensitivity. Nevertheless, none of the existing treatment strategies comprehensively target all the identified aspects. Indeed, 86.4% of the included studies primarily aim for the reduction of nasal cavity volume, while the recovery of the vascular and nerve networks, as well as of a functional airway mucosa, is completely entrusted to the body’s regenerative properties and to the ability of the implanted material to support and stimulate these regenerative processes. Within the six studies presenting alternative approaches, two targeted transitory symptom relief (W Iqbal and Gendeh, 2007; Bi and Zhou, 2016), two oversimplified ENS as merely a somatic condition, adopting antidepressants and cognitive therapy to manage avoidance behaviour (Lemogne et al., 2015; Tian et al., 2021), and the last two focused exclusively on the neuronal component, stimulating TRMP8 receptor or trigeminocervical complex (McRoberts, 2016; Le Bon et al., 2020). Similarly, the only two clinical trials registered for ENS management either evaluated botulinum toxin type A injections into the dilator nasalis muscle to increase airflow resistance (NCT00732680), or envisaged acoustic resonance therapy to break up mucus, reduce inflammation, and improve airflow (NCT07215013). Noteworthy, despite the recognized need to ensure and monitor the vascularization of implanted materials, only three studies - conducted by two scientific teams - explicated intraoperative precautions or follow-up observations addressing vascular supply. Specifically, the two groups proposed divergent strategies to promote vascular supply to the implanted graft: disrupting the submucoperiosteal layer to facilitate vessel ingrowth around the graft in cases of a bloodless plane (Houser, 2007) or preserving the integrity of the submucoperiosteal flap for ensuring a robust vascular supply (Jiang et al., 2014). Both authors reported signs of integration of implanted biomaterials within the surrounding body in terms of neovascularization or fibro-vasculature tissue within the implants. Strikingly, no ENS reconstructive approach acknowledged nerve regeneration despite the close interaction between vascular and nervous system in regulating turbinate volume, air resistance, response to environment stimuli, mucosal hydration and inflammatory responses (Smith et al., 2018; Fakoya et al., 2024). Therefore, future ENS treatments involving turbinate reconstruction should incorporate and prioritize clearly-defined strategies for vascularization and innervation to ensure proper implant integration, survival, and remodeling, ultimately restoring normal turbinate function.

## 5 Conclusion

Despite the variety of approaches tested, ENS management remains an unmet medical need. Meaningful progress toward more efficient and patient-oriented ENS management may entail the following: i) defining ENS pathophysiology by better interpretation of its underlying mechanisms; ii) stratifying patients based on type of tissue damage and biomarkers to enable the selection of customized treatments that maximize the likelihood of success; iii) standardizing outcome measures to improve comparability and clinical reliability; iv) enhancing clinical study design favouring higher-level evidence studies, such as cohort or randomized controlled trials, with adequate control group, sample size and sex balance; v) ensuring long-term follow-up with rigorous evaluation of the durability of therapeutic effects and systematic assessment of complications or adverse events; vi) proposing reconstructive strategies that simultaneously restore nasal aerodynamics, support implant innervation, vascularization, and mucosal healing; and vii) addressing the multifaceted nature of ENS through an integrated approach that supports psychological wellbeing.

## Data Availability

The raw data supporting the conclusions of this article will be made available by the authors, without undue reservation.
